# Supplementary material for: Harnessing the Diversity of Burkholderia spp. Prophages for Therapeutic Potential
Source: Cells. 2024 Feb 29;13(5):428. doi: 10.3390/cells13050428 (PMC10931425; doi:10.3390/cells13050428)
Supplement: Supplementary file 1 [file cells-13-00428-s001.zip › cells-2860733-supplementary.pdf]

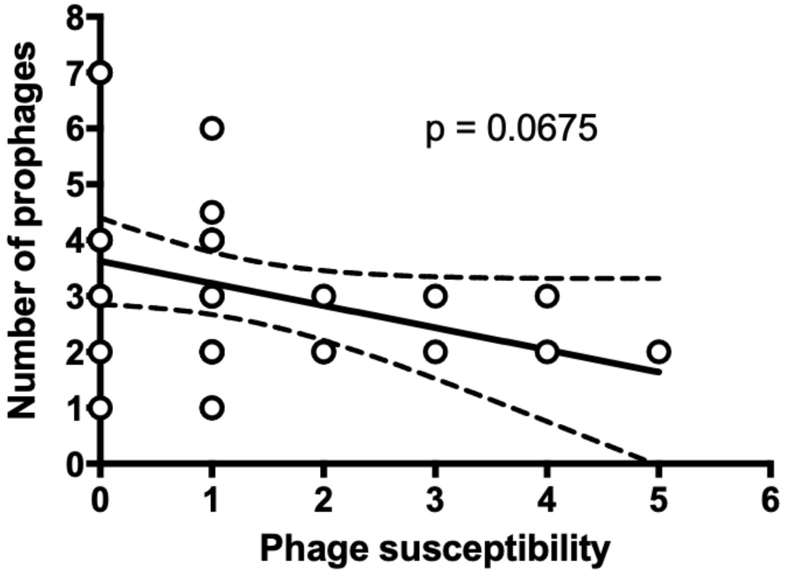

Figure S1: Prophage abundance versus phage susceptibility among 35 *Burkholderia* spp. isolates. Isolates are plotted according to the number of phages they are susceptible to (x-axis) and the number of prophages encoded in the genome (y-axis). Multiple isolates with the same coordinates are plotted on top of one another. Linear regression and 95% confidence interval are shown.

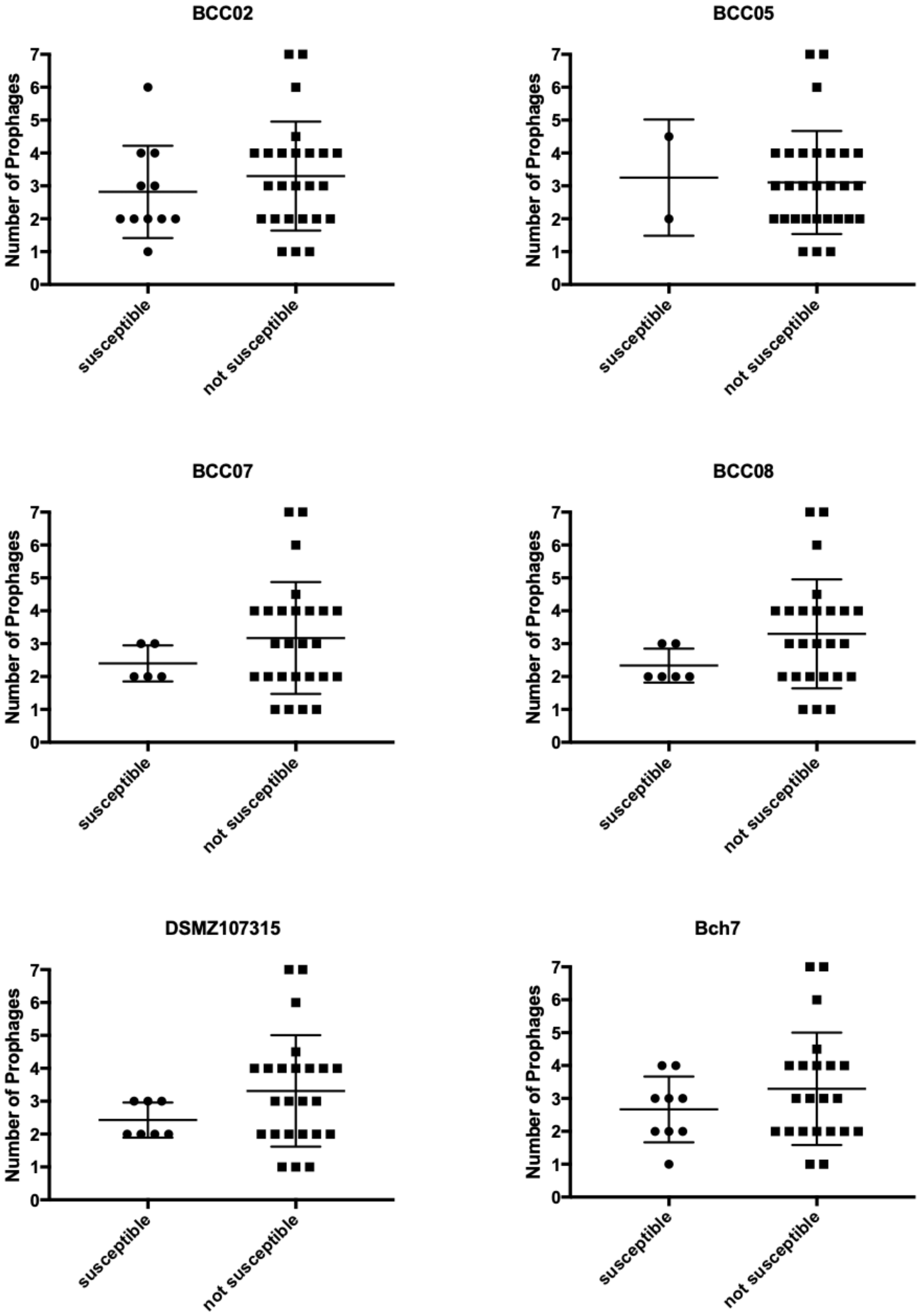

Figure S2: Prophage abundance among isolates that are or are not susceptible to each phage used in the study. Prophage abundance in isolates that were and were not susceptible to each phage. *P*-values from unpaired two-tailed t-tests were not significant in all cases.

a

	species	BCC02	BCC03	BCC04	BCC05	BCC06	BCC07	BCC08	DSMZ 107315	Bch7	Bmulti_pp1
DVT1176	B. multivorans										X
DVT1180		*	*	*							X
DVT1161								3.9			
DVT1181										6.5	
DVT1140										6.8	
DVT1171										5.8	
DVT1172										6.6	
DVT1166					*	*					X
DVT1177											X
DVT1153		4.3	4.3	4.6							X
DVT1159											X
DVT1608											X
DVT1167		2.3		2.2							
DVT1173											X
DVT1178											X
DVT1170		3.6	3.8	4.3							X

b

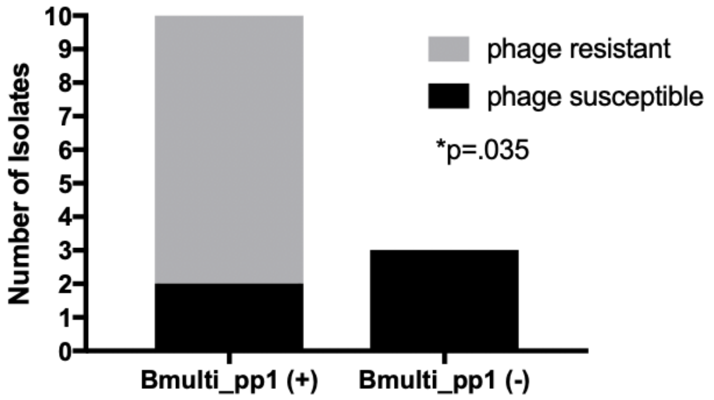

Figure S3: Prophage Bmulti_pp1 is associated with phage resistance in *B. multivorans*. (a) Phage susceptibility of 16 *B. multivorans* isolates, with isolates encoding prophage Bmulti_pp1 indicated with an X to the right of the figure. (b) Number of isolates that were resistant (grey) or susceptible (black) to any phage among isolates with (+) or without (-) prophage Bmulti_pp1. Four isolates from the same patient (grey box in panel (a)) were only counted once in this comparison. *P*-value is from Fisher's Exact Test comparing Bmulti_pp1 (+) and (-) groups.

Table S1. ***Burkholderia*** **spp. prophage-derived phages**

Phage ID	Source isolate	Source species	Target isolate	Target species
BCC02	DVT1180	*B. multivorans*	DVT1139	*B. cenocepacia*
BCC03	DVT1180	*B. multivorans*	DVT614	*B. cenocepacia*
BCC04	DVT1180	*B. multivorans*	DVT1165	*B. cenocepacia*
BCC05	DVT1166	*B. multivorans*	DVT599	*B. cenocepacia*
BCC06	DVT1166	*B. multivorans*	DVT1154	*B. cenocepacia*
BCC07	DVT1155	*B. seminalis*	DVT614	*B. cenocepacia*
BCC08	DVT1155	*B. seminalis*	DVT790	*B. cenocepacia*

Table S2. Prophages identified in *Burkholderia* spp. bacterial isolate genomes

Prophage ID	Host Isolate	Host Species	Length (bp)	% GC	Cluster	Isolated phage	Most Common Phage (# genes that match)	status
DVT1139_prophage1_contig44	DVT1139	*B. cenocepacia*	22693	69.1	Bceno_pp6	-	PHAGE_Burkho_KL3_NC_015266(24)	intact
DVT1140_prophage1_contig6	DVT1140	*B. multivorans*	39836	63.8	Bmulti_pp2 (same patient)	-	PHAGE_Burkho_KS10_NC_011216(43)	intact
DVT1140_prophage2_contig10	DVT1140	*B. multivorans*	32331	62.2	Bmulti_pp3 (same patient)	-	PHAGE_Escher_vB_EcoM_ECOO78_NC_041926(8)	intact
DVT1140_prophage3_contig29	DVT1140	*B. multivorans*	32989	65.2	Bmulti_pp4 (same patient)	-	PHAGE_Burkho_KS5_NC_015265(37)	intact
DVT1140_prophage4_contig3	DVT1140	*B. multivorans*	10969	61.9	Bmulti_pp5 (same patient)	-	PHAGE_Ralsto_RS_PII_1_NC_047804(3)	incomplete
DVT1152_prophage1_contig10	DVT1152	*B. cepacia*	36096	63.3	-	-	PHAGE_Mannhe_vB_MhM_3927AP2_NC_028766(14)	intact
DVT1152_prophage2_contig5	DVT1152	*B. cepacia*	28889	63.2	-	-	PHAGE_Escher_vB_EcoM_ECO1230_10_NC_027995(8)	incomplete
DVT1153_prophage1_contig21	DVT1153	*B. multivorans*	47703	62.2	Bmulti_pp12	-	PHAGE_Escher_vB_EcoM_ECOO78_NC_041926(8)	intact
DVT1153_prophage2_contig39	DVT1153	*B. multivorans*	34871	62.5	-	-	PHAGE_Salmon_SEN34_NC_028699(21)	questionable
DVT1153_prophage3_contig15	DVT1153	*B. multivorans*	14848	65.2	Bmulti_pp1	-	PHAGE_Burkho_KS5_NC_015265(20)	incomplete
DVT1153_prophage4_contig19	DVT1153	*B. multivorans*	4846	59.3	-	-	PHAGE_Burkho_phi1026b_NC_005284(2)	incomplete
DVT1153_prophage5_contig36	DVT1153	*B. multivorans*	21955	64.7	-	-	PHAGE_Burkho_KS5_NC_015265(18)	incomplete
DVT1153_prophage6_contig40	DVT1153	*B. multivorans*	49434	65	-	-	PHAGE_Stx2_c_1717_NC_011357(3)	incomplete
DVT1154_prophage1_contig22	DVT1154	*B. cenocepacia*	29199	68.1	Bceno_pp6 (same patient)	-	PHAGE_Burkho_phiE202_NC_009234(24)	intact
DVT1154_prophage2_contig28	DVT1154	*B. cenocepacia*	49434	65	Bceno_pp18	-	PHAGE_Salmon_SEN34_NC_028699(20)	intact
DVT1154_prophage3_contig6	DVT1154	*B. cenocepacia*	19213	65	Bceno_pp19	-	PHAGE_Burkho_BcepC6B_NC_005887(5)	incomplete
DVT1155_prophage1_contig4	DVT1155	*B. seminalis*	44646	62.9	-	-	PHAGE_Rhodof_P26218_NC_029061(7)	intact
DVT1155_prophage2_contig10	DVT1155	*B. seminalis*	38090	64.9	-	BCC07	PHAGE_Burkho_AP3_NC_047752(38)	intact
DVT1155_prophage3_contig20	DVT1155	*B. seminalis*	37802	63.6	-	BCC08	PHAGE_Burkho_KL3_NC_015266(39)	intact
DVT1155_prophage4_contig21	DVT1155	*B. seminalis*	37166	61.8	-	-	PHAGE_Burkho_ST79_NC_021343(41)	intact
DVT1156_prophage1_contig3	DVT1156	*B. cenocepacia*	7515	68	Bceno_pp10	-	PHAGE_Escher_phAPEC8_NC_020079(4)	incomplete
DVT1156_prophage2_contig35	DVT1156	*B. cenocepacia*	10085	64.2	-	-	PHAGE_Burkho_phi6442_NC_009235(3)	incomplete
DVT1156_prophage3_contig70	DVT1156	*B. cenocepacia*	11199	59.3	-	-	PHAGE_Rhizob_RR1_A_NC_021560(1)	incomplete
DVT1156_prophage4_contig71	DVT1156	*B. cenocepacia*	8814	66.7	Bceno_pp9	-	PHAGE_Cellul_phi38:1_NC_021796(1)	incomplete
DVT1157_prophage1_contig11	DVT1157	*B. vietnamensis*	47046	65.5	-	-	PHAGE_Burkho_BcepC6B_NC_005887(37)	intact
DVT1157_prophage2_contig18	DVT1157	*B. vietnamensis*	41263	61.5	-	-	PHAGE_Burkho_Bcep176_NC_007497(24)	intact
DVT1157_prophage3_contig21	DVT1157	*B. vietnamensis*	25312	63.2	-	-	PHAGE_Escher_vB_EcoM_ECOO78_NC_041926(9)	incomplete
DVT1159_prophage1_contig37	DVT1159	*B. multivorans*	37188	63.4	Bmulti_pp1	-	PHAGE_Burkho_KS5_NC_015265(35)	intact
DVT1160_prophage1_contig26	DVT1160	*B. cenocepacia*	7515	68	Bceno_pp10	-	PHAGE_Escher_ESCO5_NC_047776(4)	incomplete
DVT1160_prophage2_contig71	DVT1160	*B. cenocepacia*	8810	66.7	Bceno_pp9	-	PHAGE_Klebsi_ST437_OXA245phi4.1_NC_049448(1)	incomplete
DVT1161_prophage1_contig22	DVT1161	*B. multivorans*	38405	62.3	Bmulti_pp12	-	PHAGE_Escher_vB_EcoM_ECO1230_10_NC_027995(8)	questionable
DVT1161_prophage2_contig8	DVT1161	*B. multivorans*	17709	64.3	Bmulti_pp11	-	PHAGE_Pectob_CBB_NC_041878(2)	incomplete
DVT1163_prophage1_contig12	DVT1163	*B. cenocepacia*	44021	65	Bceno_pp16	-	PHAGE_Salmon_SEN34_NC_028699(14)	intact
DVT1163_prophage2_contig25	DVT1163	*B. cenocepacia*	12189	61.2	Bceno_pp17	-	PHAGE_Salmon_SEN34_NC_028699(3)	incomplete
DVT1165_prophage1_contig8	DVT1165	*B. cenocepacia*	34706	63.3	-	-	PHAGE_Burkho_BcepMu_NC_005882(46)	intact
DVT1165_prophage2_contig39	DVT1165	*B. cenocepacia*	30623	65.5	-	-	PHAGE_Myxoco_Mx8_NC_003085(7)	intact
DVT1165_prophage3_contig41	DVT1165	*B. cenocepacia*	40032	63.5	-	-	PHAGE_Burkho_KS10_NC_011216(43)	intact
DVT1165_prophage4_contig19	DVT1165	*B. cenocepacia*	33431	62.5	-	-	PHAGE_Pseudo_PAJU2_NC_011373(4)	incomplete
DVT1166_prophage1_contig10	DVT1166	*B. multivorans*	29994	63	-	BCC05/BCC06	PHAGE_Burkho_phiE12_2_NC_009236(25)	intact
DVT1166_prophage2_contig19	DVT1166	*B. multivorans*	23631	65.3	-	-	PHAGE_Salmon_SEN34_NC_028699(23)	intact
DVT1166_prophage3_contig12	DVT1166	*B. multivorans*	8333	64.8	-	-	PHAGE_Bacill_vB_BtS_BMBtp14_NC_048640(2)	incomplete
DVT1166_prophage4_contig19	DVT1166	*B. multivorans*	19712	61.2	-	-	PHAGE_Burkho_phi1026b_NC_005284(3)	incomplete
DVT1166_prophage5_contig21	DVT1166	*B. multivorans*	18687	64.8	-	-	PHAGE_Burkho_KS5_NC_015265(9)	incomplete
DVT1166_prophage6_contig38	DVT1166	*B. multivorans*	22687	64.3	Bmulti_pp1	-	PHAGE_Burkho_KS5_NC_015265(11)	incomplete
DVT1166_prophage7_contig45	DVT1166	*B. multivorans*	11827	58.2	-	-	PHAGE_Burkho_phiE12_2_NC_009236(3)	incomplete
DVT1167_prophage1_contig8	DVT1167	*B. multivorans*	62320	62.7	-	-	PHAGE_Escher_vB_EcoM_ECOO78_NC_041926(9)	questionable
DVT1167_prophage2_contig16	DVT1167	*B. multivorans*	12701	62	-	-	PHAGE_Salmon_SEN34_NC_028699(5)	incomplete
DVT1170_prophage1_contig24	DVT1170	*B. multivorans*	41030	64.4	Bmulti_pp1	-	PHAGE_Burkho_KS5_NC_015265(35)	intact
DVT1170_prophage2_contig27	DVT1170	*B. multivorans*	13392	64.4	Bmulti_pp13	-	PHAGE_Erwini_PEp14_NC_016767(3)	incomplete
DVT1170_prophage3_contig32	DVT1170	*B. multivorans*	20473	63.7	Bmulti_pp14	-	PHAGE_Burkho_phiE125_NC_003309(3)	incomplete
DVT1170_prophage4_contig53	DVT1170	*B. multivorans*	31843	63.8	Bmulti_pp15	-	PHAGE_Burkho_vB_BmuP_KL4_NC_047958(11)	incomplete
DVT1171_prophage1_contig9	DVT1171	*B. multivorans*	39836	63.8	Bmulti_pp2 (same patient)	-	PHAGE_Burkho_KS10_NC_011216(43)	intact
DVT1171_prophage2_contig20	DVT1171	*B. multivorans*	32331	62.2	Bmulti_pp3 (same patient)	-	PHAGE_Escher_vB_EcoM_ECO1230_10_NC_027995(7)	intact
DVT1171_prophage3_contig43	DVT1171	*B. multivorans*	33430	65.2	Bmulti_pp4 (same patient)	-	PHAGE_Burkho_KS5_NC_015265(37)	intact
DVT1171_prophage4_contig6	DVT1171	*B. multivorans*	12231	60.8	Bmulti_pp5 (same patient)	-	PHAGE_Ralsto_RS_PII_1_NC_047804(3)	incomplete
DVT1172_prophage1_contig10	DVT1172	*B. multivorans*	39836	63.8	Bmulti_pp2 (same patient)	-	PHAGE_Burkho_KS10_NC_011216(43)	intact
DVT1172_prophage2_contig11	DVT1172	*B. multivorans*	32331	62.2	Bmulti_pp3 (same patient)	-	PHAGE_Escher_vB_EcoM_ECOO78_NC_041926(7)	questionable
DVT1172_prophage3_contig41	DVT1172	*B. multivorans*	32970	65.2	Bmulti_pp4 (same patient)	-	PHAGE_Burkho_KS5_NC_015265(38)	intact
DVT1172_prophage4_contig15	DVT1172	*B. multivorans*	10969	61.9	Bmulti_pp5 (same patient)	-	PHAGE_Ralsto_RS_PII_1_NC_047804(3)	incomplete
DVT1173_prophage1_contig22	DVT1173	*B. multivorans*	17089	62.4	-	-	PHAGE_Entero_SfV_NC_003444(4)	intact
DVT1173_prophage2_contig23	DVT1173	*B. multivorans*	37187	63.3	Bmulti_pp1	-	PHAGE_Burkho_KS5_NC_015265(35)	intact
DVT1173_prophage3_contig22	DVT1173	*B. multivorans*	16888	62.3	-	-	PHAGE_Burkho_phi1026b_NC_005284(2)	incomplete
DVT1174_prophage1_contig4	DVT1174	*B. cenocepacia*	40429	62.4	-	-	PHAGE_Escher_vB_EcoM_ECOO78_NC_041926(9)	questionable
DVT1174_prophage2_contig43	DVT1174	*B. cenocepacia*	16336	62.1	-	-	PHAGE_Entero_fiAA91_ss_NC_022750(2)	incomplete
DVT1175_prophage1_contig50	DVT1175	*B. cenocepacia*	8307	63.1	Bceno_pp20	-	PHAGE_Stx2_c_1717_NC_011357(3)	questionable
DVT1175_prophage2_contig72	DVT1175	*B. cenocepacia*	16371	62.3	Bceno_pp7	-	PHAGE_Burkho_KS9_NC_013055(21)	intact
DVT1175_prophage3_contig78	DVT1175	*B. cenocepacia*	18522	62.4	Bceno_pp8	-	PHAGE_Burkho_KS9_NC_013055(14)	questionable
DVT1176_prophage1_contig17	DVT1176	*B. multivorans*	44249	62.3	-	-	PHAGE_Burkho_Bcep176_NC_007497(35)	incomplete
DVT1176_prophage2_contig30	DVT1176	*B. multivorans*	22884	62.5	-	-	PHAGE_Salmon_SEN34_NC_028699(3)	incomplete
DVT1176_prophage3_contig35	DVT1176	*B. multivorans*	26076	64.5	Bmulti_pp1	-	PHAGE_Burkho_KS5_NC_015265(16)	incomplete
DVT1176_prophage4_contig60	DVT1176	*B. multivorans*	14004	65.5	Bmulti_pp1	-	PHAGE_Burkho_KS5_NC_015265(17)	incomplete
DVT1177_prophage1_contig16	DVT1177	*B. multivorans*	37188	63.4	Bmulti_pp1	-	PHAGE_Burkho_KS5_NC_015265(35)	intact
DVT1177_prophage2_contig30	DVT1177	*B. multivorans*	9446	61.6	-	-	PHAGE_Escher_SH2026Stx1_NC_049919(3)	incomplete
DVT1178_prophage1_contig21	DVT1178	*B. multivorans*	39737	64	Bmulti_pp1	-	PHAGE_Burkho_KS5_NC_015265(35)	intact
DVT1178_prophage2_contig27	DVT1178	*B. multivorans*	20473	63.7	Bmulti_pp14	-	PHAGE_Burkho_phiE125_NC_003309(3)	incomplete
DVT1178_prophage3_contig29	DVT1178	*B. multivorans*	13392	64.4	Bmulti_pp13	-	PHAGE_Serrat_Parlo_NC_048758(3)	incomplete
DVT1178_prophage4_contig96	DVT1178	*B. multivorans*	20455	61.2	Bmulti_pp15	-	PHAGE_Burkho_vB_BmuP_KL4_NC_047958(12)	incomplete
DVT1179_prophage1_contig22	DVT1179	*B. cenocepacia*	44021	65	Bceno_pp16	-	PHAGE_Salmon_SEN34_NC_028699(14)	intact
DVT1179_prophage2_contig129	DVT1179	*B. cenocepacia*	7338	59.8	-	-	PHAGE_Stx2_c_1717_NC_011357(3)	questionable
DVT1179_prophage3_contig91	DVT1179	*B. cenocepacia*	12189	61.2	Bceno_pp17	-	PHAGE_Salmon_SEN34_NC_028699(3)	incomplete
DVT1180_prophage1_contig3	DVT1180	*B. multivorans*	34935	64.2	-	-	PHAGE_Pseudo_NP1_NC_031058(5)	intact
DVT1180_prophage2_contig53	DVT1180	*B. multivorans*	17357	60.8	-	-	PHAGE_Salmon_118970_sal3_NC_031940(7)	intact
DVT1180_prophage3_contig50	DVT1180	*B. multivorans*	12911	66.6	-	BCC02/BCC03/BCC04	PHAGE_Burkho_KS5_NC_015265(19)	incomplete
DVT1180_prophage4_contig53	DVT1180	*B. multivorans*	18839	60.6	-	-	PHAGE_Burkho_Bcep176_NC_007497(9)	incomplete
DVT1180_prophage5_contig85	DVT1180	*B. multivorans*	19107	65.1	-	BCC02/BCC03/BCC04	PHAGE_Burkho_KS5_NC_015265(15)	incomplete
DVT1180_prophage6_contig113	DVT1180	*B. multivorans*	13341	65.3	Bmulti_pp1	-	PHAGE_Burkho_KS5_NC_015265(19)	incomplete
DVT1180_prophage7_contig120	DVT1180	*B. multivorans*	13133	63.8	-	-	PHAGE_Burkho_KS5_NC_015265(12)	incomplete
DVT1181_prophage1_contig6	DVT1181	*B. multivorans*	39836	63.8	Bmulti_pp2 (same patient)	-	PHAGE_Burkho_KS10_NC_011216(43)	intact
DVT1181_prophage2_contig16	DVT1181	*B. multivorans*	32331	62.2	Bmulti_pp3 (same patient)	-	PHAGE_Escher_vB_EcoM_ECOO78_NC_041926(7)	intact
DVT1181_prophage3_contig35	DVT1181	*B. multivorans*	32989	65.2	Bmulti_pp4 (same patient)	-	PHAGE_Burkho_KS5_NC_015265(37)	intact
DVT1181_prophage4_contig8	DVT1181	*B. multivorans*	12231	60.8	Bmulti_pp5 (same patient)	-	PHAGE_Ralsto_RS_PI_1_NC_047816(3)	incomplete
DVT1600_prophage1_contig13	DVT1600	*B. gladiolii*	38172	61.2	-	-	PHAGE_Burkho_KS9_NC_013055(22)	intact
DVT1600_prophage2_contig18	DVT1600	*B. gladiolii*	34696	61.3	-	-	PHAGE_Sphing_Lacusarx_NC_041927(4)	intact
DVT1600_prophage3_contig22	DVT1600	*B. gladiolii*	39615	64.3	-	-	PHAGE_Burkho_AP3_NC_047752(35)	intact
DVT1608_prophage1_contig6	DVT1608	*B. multivorans*	50362	62.9	-	-	PHAGE_Aeromo_vB_AsaM_56_NC_019527(15)	intact
DVT1608_prophage2_contig19	DVT1608	*B. multivorans*	37188	63.4	Bmulti_pp1	-	PHAGE_Burkho_KS5_NC_015265(35)	intact
DVT1608_prophage3_contig1	DVT1608	*B. multivorans*	18352	64.6	Bmulti_pp11	-	PHAGE_Pectob_CBB_NC_041878(2)	incomplete
DVT1608_prophage4_contig31	DVT1608	*B. multivorans*	23671	60.5	-	-	PHAGE_Burkho_vB_BmuP_KL4_NC_047958(9)	incomplete
DVT1627_prophage1_contig27	DVT1627	*B. cenocepacia*	34069	62.4	Bceno_pp7	-	PHAGE_Burkho_KS9_NC_013055(32)	intact
DVT599_prophage1_contig3	DVT599	*B. cenocepacia*	26389	65.4	Bceno_pp18	-	PHAGE_Salmon_SEN34_NC_028699(19)	questionable
DVT599_prophage2_contig15	DVT599	*B. cenocepacia*	29200	68.2	Bceno_pp6 (same patient)	-	PHAGE_Burkho_KL3_NC_015266(24)	intact
DVT599_prophage3_contig3	DVT599	*B. cenocepacia*	23915	66.1	-	-	PHAGE_Salmon_118970_sal3_NC_031940(2)	incomplete
DVT599_prophage4_contig4	DVT599	*B. cenocepacia*	13641	61.6	-	-	PHAGE_Ralsto_RsoM1USA_NC_049432(2)	incomplete
DVT599_prophage5_contig25	DVT599	*B. cenocepacia*	10967	60.4	-	-	PHAGE_Burkho_vB_BmuP_KL4_NC_047958(4)	incomplete
DVT599_prophage6_contig25	DVT599	*B. cenocepacia*	22203	65.2	Bceno_pp19	-	PHAGE_Burkho_BcepC6B_NC_005887(5)	incomplete
DVT613_prophage1_contig9	DVT613	*B. pseudomultivorans*	8119	71.2	-	-	PHAGE_Bacill_G_NC_023719(2)	incomplete
DVT613_prophage2_contig45	DVT613	*B. pseudomultivorans*	7720	66.4	-	-	PHAGE_Plankt_PaV_LD_NC_016564(1)	incomplete
DVT613_prophage3_contig100	DVT613	*B. pseudomultivorans*	9479	59.1	-	-	PHAGE_Plankt_PaV_LD_NC_016564(1)	incomplete
DVT614_prophage1_contig13	DVT614	*B. cenocepacia*	18756	62.7	Bceno_pp8	-	PHAGE_Burkho_KS9_NC_013055(14)	questionable
DVT614_prophage2_contig84	DVT614	*B. cenocepacia*	14871	61.7	Bceno_pp7	-	PHAGE_Burkho_KS9_NC_013055(20)	questionable
DVT753_prophage1_contig2	DVT753	*B. cenocepacia*	7515	68.1	Bceno_pp10	-	PHAGE_Escher_phAPEC8_NC_020079(4)	incomplete
DVT753_prophage2_contig40	DVT753	*B. cenocepacia*	11919	67.5	Bceno_pp9	-	PHAGE_Bacill_SP_15_NC_031245(1)	incomplete
DVT790_prophage1_contig50	DVT790	*B. cenocepacia*	8265	63.1	Bceno_pp20	-	PHAGE_Stx2_c_Stx2a_F451_NC_049924(3)	questionable
DVT790_prophage2_contig94	DVT790	*B. cenocepacia*	18495	62.4	Bceno_pp8	-	PHAGE_Burkho_KS9_NC_013055(14)	questionable
